# Reconstructing neural circuits using multiresolution correlated light and electron microscopy

**DOI:** 10.3389/fncir.2022.753496

**Published:** 2022-10-21

**Authors:** Karl Friedrichsen, Pratyush Ramakrishna, Jen-Chun Hsiang, Katia Valkova, Daniel Kerschensteiner, Josh L. Morgan

**Affiliations:** ^1^Department of Ophthalmology and Visual Sciences, Washington University in St. Louis, St. Louis, MO, United States; ^2^Department of Neuroscience, Washington University in St. Louis, St. Louis, MO, United States; ^3^Department of Biomedical Engineering, Washington University in St. Louis, St. Louis, MO, United States

**Keywords:** connectomics, neural circuit, correlated light and electron microscopy (CLEM), synapse, confocal 3D microscopy, tissue mapping, electron microscopy

## Abstract

Correlated light and electron microscopy (CLEM) can be used to combine functional and molecular characterizations of neurons with detailed anatomical maps of their synaptic organization. Here we describe a multiresolution approach to CLEM (mrCLEM) that efficiently targets electron microscopy (EM) imaging to optically characterized cells while maintaining optimal tissue preparation for high-throughput EM reconstruction. This approach hinges on the ease with which arrays of sections collected on a solid substrate can be repeatedly imaged at different scales using scanning electron microscopy. We match this multiresolution EM imaging with multiresolution confocal mapping of the aldehyde-fixed tissue. Features visible in lower resolution EM correspond well to features visible in densely labeled optical maps of fixed tissue. Iterative feature matching, starting with gross anatomical correspondences and ending with subcellular structure, can then be used to target high-resolution EM image acquisition and annotation to cells of interest. To demonstrate this technique and range of images used to link live optical imaging to EM reconstructions, we provide a walkthrough of a mouse retinal light to EM experiment as well as some examples from mouse brain slices.

## Introduction

Electron microscopy (EM) can reveal the complete nanoscale arrangement of cells and organelles in a piece of tissue. Cells and subcellular structures are distinguished not by selective labeling, but by brute resolving power. Three dimensional EM (3DEM) is therefore ideal for mapping the dense networks of fine neurites and synaptic connections that delineate the flow of information through nervous tissue (Morgan and Lichtman, 2013). While this technique has been the gold standard for describing the synaptic connectivity of neurons for more than 60 years (Sjostrand, 1958), obtaining the thousands of images required to reconstruct the synaptic connectivity of a significant proportion of even one neuron was extremely labor intensive (Hamos et al., 1985; White et al., 1986; Freed and Sterling, 1988; Famiglietti, 1991). Advances in EM tissue processing, imaging, and data management have made it feasible to digitize large volumes of neural tissue (3DEM) and reconstruct their circuitry (Jain et al., 2010; Turaga et al., 2010; Bock et al., 2011; Helmstaedter et al., 2013; Hayworth et al., 2014; Kasthuri et al., 2015; Morgan et al., 2016; Morgan and Lichtman, 2017; Januszewski et al., 2018; Yin et al., 2020).

Chief among the tissue processing advances is automatic sectioning. Currently, the most common automated approach to sectioning is blockface EM where the surface of tissue block is removed with a diamond knife or focused ion beam so that the underlaying tissue is exposed for imaging with scanning electron microscopy(SEM) (Briggman et al., 2011). Sequential surface removal and imaging produces 3DEM volumes. Not having to deal with ultrathin sections eliminates many of challenges of traditional serial section electron microscopy, but it also eliminates the option to reimage tissue as the EM reconstruction provides more information about the tissue. An alternative approach, and the one applied here, is the automated collection of sections. The automated tape collecting ultramicrotome (ATUM) adds a conveyor belt to the back of the diamond knife waterboat in which ultrathin sections are traditionally collected (Schalek et al., 2012; Hayworth et al., 2014). This conveyor belt, picks sections up as they are cut so that thousands of ultrathin sections can be cut and collected without stopping and starting the microtome and without manual intervention. Sections collected onto tape can be imaged either with SEM or transmission electron microscopy (TEM).

The primary limitations in studying the nervous system with 3DEM are: (1) Image volume size is limited by the long acquisition times, large data sizes, and long analysis times demanded by high pixel densities. (2) The tissue is fixed, metalized, plasticized, and sectioned so live imaging is not an option. (3) Labeling specific molecules, usually by antibody labeling, is challenging due to the heavy fixation and staining required for high-throughput EM. The heavy osmium staining provides enough signal for fast imaging but interferes with antibody binding. These limitations can be overcome by first imaging a piece of neural tissue optically and then reconstructing targeted regions of the same tissue by 3DEM (Bock et al., 2011); a technique called correlative light electron microscopy (CLEM) (de Boer et al., 2015). Optical data can provide dynamic morphological, physiological, and molecular characterizations of neurons that can then be mapped onto high-resolution EM anatomical reconstructions.

A major challenge of CLEM is targeting; directing high-resolution EM image acquisition to the optically characterized region of the tissue and then locating optically characterized neurons or synapses within the EM volume. In EM techniques in which the tissue is destroyed during imaging (blockface scanning) care must be taken to identify landmarks during the single pass of cutting that can be used to direct high resolution imaging. In techniques where sections are preserved and imaged (serial section EM), targeting can be labor intensive because landmarks must be identified in two dimensional images and extrapolated across thousands of sections (Morgan et al., 2016).

One approach to CLEM targeting is to label tissue with a stain that is visible in both light and electron micrographs. Most of these approaches involve labeling a cell with something that will catalyze the oxidation of a diaminobenzidine (DAB) solution to produce precipitate that is visible both optically and in tissue processed for electron microscopy. The approach commonly used in electrophysiology is to label a cell by filling its recording electrode with horseradish peroxidase (HRP) (Robson et al., 1978). Horseradish peroxidase (HRP) can also be bound to antibodies to label specific proteins (Trachtenberg et al., 2002). Transgenic approaches to driving the DAB reaction [mHRP (Li et al., 2010), miniSog (Shu et al., 2011), or APEX (Martell et al., 2012; Lam et al., 2015; Zhang et al., 2019)] can produce optical and EM labels targeted to specific cell types or organelles. A wide range of fluorescent dyes can also drive the DAB reaction directly through photoconversion (Maranto, 1982). One of the limitations of the DAB approach to CLEM is that the DAB precipitate can obscure ultrastructural detail. Labeling native proteins or transgenic labels with electron-dense nanogold conjugated antibodies avoids the precipitate (Faulk and Taylor, 1971), but traditionally requires membrane permeabilization for non-surface labeling (see Fulton and Briggman, 2021 for alternative). Light and EM visible fiducials can also be introduced with the optical microscope itself in the case of near-infrared branding (NIRB) (Bishop et al., 2011). The collection of electron-dense optical label approaches is varied, but suffer from the limitation that optimal tissue processing for generating the EM label diverges from the optimal tissue parameters for high-contrast/high-throughput 3DEM reconstruction of intact neuronal circuits.

A second approach for matching light and EM is to optically map ubiquitous biological features that can be recognized in an electron micrograph by their pattern (pattern matching vs. label matching). Blood vessels and cell nuclei are attractive targets for this mapping as they are easy to label with fluorescent stains, are visible in any 3DEM preparation, and span pattern matching scales from centimeters to micrometers (Vishwanathan et al., 2017). Specific labeling and imaging of these features was successful in previous large-scale reconstructions of functionally characterized neural circuits (Bock et al., 2011). A principal challenge of this pattern matching approach is the difficulty in acquiring and annotating EM images that can be matched to optical images across a range of scales (millimeter to nanometer). Large EM fields of view are required for matching blood vessel morphology, while high-resolution images are required for reconstructing and matching neurite morphology. Furthermore, if optical images, EM images, and matching features to not all lay in the same plane, then 3D reconstruction of light and EM volumes may be required before correspondence can be identified.

Multi-shot EM imaging approaches allow for the same sections to be imaged multiple times at different resolutions and then matched to complimentary sets of optical images (Schifferer et al., 2021). Here we describe a multiresolution CLEM (mrCLEM) workflow, which combines 3D confocal maps of fixed tissue with automated-tape collecting ultramicrotomy (ATUM). ATUM preserves large numbers of ultrathin (∼30–40 nm) sections on a stable substrate and is, therefore, uniquely suited for repeated imaging and mrCLEM (Hirabayashi et al., 2018; Snaidero et al., 2020; Schifferer et al., 2021). By combining ATUM with confocal mapping of dense features, we can first match the vasculature in a ∼1000-nm resolution 3DEM volume to the vasculature of optical images of the same tissue, then match cell bodies and large neurites in a ∼20-nm resolution 3DEM volume, and finally match fine neurites in an embedded 4-nm resolution 3DEM volume. In our workflow, we use 2-photon imaging for live imaging (penetration and repeated imaging without out-of-focus bleaching) and confocal imaging for mapping reference features in fixed tissue (4 + channel imaging, spectral separation, reflected light).

Below, we provide a guide to this approach as well as examples from mouse thalamus and mouse retina. To demonstrate the range of scale and modalities applied to a single piece of tissue, we walk through one CLEM experiment from beginning. In this experiment, the response properties of the neurites of retinal amacrine cells are characterized with live 2-photon calcium imaging. The same neurites are then targeted for 3DEM circuit reconstruction. We also provide examples multiresolution CLEM is used in brain slices where 3DEM is used to examine the synaptic connectivity of retinal ganglion cell axons that have been fluorescently labeled according to whether they originate from the left or right eye.

## Materials and equipment

### Optical microscopy reagents

4’,6-diamidino-2-phenylindole (DAPI) (Invitrogen D1306), Sulforhodamine (Chemodex S0025), FluoroMyelin (Invitrogen F34652).

### Electron microscopy reagents

Paraformaldehyde (EMS, 16714), glutaraldehyde (EMS 50-262-17), calcium chloride (VWR BDH7308-1), phosphate buffer saline (Genesee Scientific 25-507B), sodium cacodylate (Fisher Scientific International 50-366-664), osmium tetroxide (Fisher Scientific Inc 50-332-09), ferricyanide (EMS 20150), pyrogallol (SIGMA 16040), thiocarbohydrazide (EMS 21900), maleate buffer (EMS 11730-08), uranyl acetate (Fisher Scientific International 22400-4), lead nitrate (EMSURE 10099-74-8), aspartic acid (SIGMA-Aldrich A93100), acetonitrile (Fisher Scientific International 50-980-146), Spurrs (EMS 14300), EMbed 812 (EMS 14121).

### Tissue preparation

For functional imaging, mouse retinas were isolated *via* dissection under infrared binocular stereo microscope. The retinas were immersed in mouse artificial cerebrospinal (mACSF) fluid buffered with sodium bicarbonate throughout the dissection and imaging. mACSF_NaHCO3_ contained (in mM) 125 NaCl, 2.5 KCl, 1 MgCl_2_, 1.25 NaH_2_PO_4_, 2 CaCl_2_, 20 glucose, 26 NaHCO_3_ and 0.5 L-Glutamine equilibrated with 95% O_2_/5% CO_2_. Retinas were flat mounted on transparent membrane disks (Anodisc 13, Whatman, Maidstone, United Kingdom, for two-photon imaging) or membrane disks (HABGO1300, MilliporeSigma, Burlington, MA, United States, for confocal imaging).

For obtaining fixed dorsal lateral geniculate nucleus (dLGNs) of the thalamus, mice were transcardially perfused using a NE-1000 Single Syringe Pump (SyringePump.com, Farmingdale, NY, United States) (Morgan et al., 2016). Sections were cut using a Compresstome vibratome (Precisionary VF-200-0Z).

### Two-photon microscope

Two-photon images were acquired with a custom-built upright two-photon microscope (Scientifica, Uckfield, United Kingdom) and a Mai-Tai laser (Spectra-Physics, Santa Clara, CA, United States). The microscope is controlled by the Scanimage r3.8 MATLAB toolbox (Pologruto et al., 2003).

### Confocal microscope (for optical correlation mapping)

We acquired confocal micrographs with an Olympus FV300 and an Olympus FV1000. The flexible emission filter settings of the FV1000 are helpful for imaging reflected light and autofluorescence. To image reflected light it is necessary to be able to adjust (or remove) emission filters so that light of the laser’s wavelength can reach the detectors. For imaging autofluorescence, it is helpful to be able to set emission filters to capture as much light as possible while excluding the laser lines.

### Automated tape collecting ultramicrotome powertome

Automated tape collecting ultramicrotome is a reel-to-reel conveyor belt that fits in the boat of an ultramicrotomy diamond knife (Schalek et al., 2012; Hayworth et al., 2014; Baena et al., 2019). Ultrathin sections float across the water’s surface in the diamond knife boat and are either directly deposited on the conveyor belt as they are cut or are pushed onto the tape by the subsequent section. The ATUM allows for the continuous collection of sections in an isolation chamber, making it easier to collect long series of ultrathin sections (30–45 nm) with minimal section loss. This technology can be used to collect sections for both scanning electron microscopy and transmission electron microscopy. For scanning electron microscopy, we collect sections on conductive Kapton tape (Sheldahl). The conductive tape used in the datasets here was 8 mm wide Kapton tape coated with carbon in the laboratory of Jeff Lichtman or aluminum (Sheldahl, Northfield, MN, United States). Excellent performance can be obtained using carbon nanotube tape (Kubota et al., 2018) commercially available through RMC Boeckeler (Tucson, AZ, United States). Collecting sections on tape for scanning electron microscopy preserves sections on a stable substrate that can be reimaged many times over many years (Hildebrand et al., 2017). For transmission electron microscopy, sections can be collected on tape that includes electron-lucent film-filled holes (Graham et al., 2019).

### Ultramicrotome

An RMC Powertome is sold in conjunction with the RMC ATUM. The ATUM is also compatible with other microtomes. We used a Leica UC7 ultramicrotome for most of the work presented here. We trimmed section blocks with glass knives and a 90 degree or 20 degree Diatome (Hatfield, PA, United States) trimming diamond knife. We used Diatome Ultra 45 and 35 knives for ultrathin sectioning. After sectioning, we cut the collection tape and mounted it onto conductive 4-inch diameter silicon wafers (University Wafer, South Boston, MA, United States) using conductive carbon adhesive tape (EMS, Hatfield, PA, United States).

### Zeiss Merlin scanning electron microscope

The Merlin is a single beam field-emission scanning electron microscope (SEM). It is particularly well suited to multiresolution SEM as it allows for more than an order of magnitude range of electron currents, voltages, depths of fields, and working distances (see Table 1). For instance, we mapped 4-inch wafers at low resolution using 10 mm wide field of view (FOV) mosaic tiles with high current (giving high signal, low resolution) and long working distances (wide FOV, low resolution) in high depth of field mode (high depth of field, low resolution) (Table 1). Once the target is identified, the small electron beam spot sizes (<1 – 20 nm depending on conditions) that are required for high-resolution imaging are obtained by reducing current and working distance and switching to high-resolution or analytic mode. We also use an Ibss plasma asher (Burlingame, CA, United States) to plasma treat sections prior to imaging. This process increases sample contrast by etching plastic and removes surface contaminants that could cause burning during imaging (Morgan et al., 2016).

**TABLE 1:** Imaging conditions used for optical and EM imaging.

Image	Objective	FOV Size (μm)	Voxel size (μm)	Excitation wavelength (nm) electron voltage (keV)	Approximate Acquisition time	Dwell time (μs × Kalman)
Live Two-photon functional imaging	60 × 1.35 nA	*X* = 100 *Y* = 13 *Z* = ∼	*X* = 0.4 *Y* = 0.4 *Z* = ∼	933 nm	49 min	2
Live Two-photon structural imaging (fluorescence and transmitted)	60 × 1.35 nA	*X* = 100 *Y* = 100 *Z* = 40	*X* = 0.2 *Y* = 0.2 *Z* = 0.2	933 nm	40 min	2
Live Two-photon tissue mapping (fluorescence and transmitted)	60 × 1.35 nA	X = 300 Y = 300 Z = ∼50	*X* = 0.6 *Y* = 0.6 *Z* = 0.4	933 nm	40 min	2
Fixed optical confocal 10x map	10 × 0.4 na	*X* = 1270 *Y* = 1270 *Z* = ∼	*X* = 0.600 *Y* = 0.6 *Z* = ∼	405 nm, 488 nm, 635 nm	30 min	2
Fixed optical confocal 20x map	20 × 0.8 na	*X* = 635 *Y* = 636 *Z* = 96	*X* = 0.300 *Y* = 0.3 *Z* = 0.12	405 nm, 488 nm, 635 nm	1 h	2 × 4
Fixed confocal 60x map, intrinsic signals	60 × 1.35 na	*X* = 212 *Y* = 212 *Z* = 60	*X* = 0.100 *Y* = 0.1 *Z* = 0.3	405 nm, 488 nm, 635 nm	2 h	2 × 4
Fixed confocal 60x map, Sulforhodamine + DAPI	60 × 1.35 na	*X* = 212 *Y* = 212 *Z* = 60	*X* = 0.100 *Y* = 0.1 *Z* = 0.3	405 nm, 488 nm, 543 nm	2 h	2 × 4
EM overview images BSD or SE2 detector	37x	*X* = 2000 Y = 2000 *Z* = 42	*X* = 0.600 *Y* = 0.6 *Z* = 0.04	8 keV	7.5 h	1
EM medium resolution BSD (targeting)	2500x	*X* = 164 *Y* = 164 *Z* = ∼	*X* = 0.0440 *Y* = 0.04 *Z* = 0.04	8 keV	17 h	0.3
EM High resolution	1900x	*X* = 120 *Y* = 120 *Z* = 42	*X* = 0.004 *Y* = 0.004 *Z* = 0.04	1 keV	6 weeks	0.2–0.4
EM medium resolution In Lens (large neurite reconstruction)	1500x	*X* = 630 *Y* = 630 *Z* = 34	*X* = 0.010 *Y* = 0.01 *Z* = 0.04	1 keV	3 weeks	0.1–0.2
EM medium resolution BSD (large neurite reconstruction)	350x	*X* = 630 *Y* = 630 *Z* = 8	*X* = 0.0110 *Y* = 0.01 *Z* = 0.04	8 keV	1 week	1

To maximize resolution and prevent photo bleaching, we use low laser power and short dwell times for confocal imaging and then use Kalman averaging (2–6 repeats) to reduce noise. In fixed tissue with limited bleaching (GFP variants and Alexa dyes) we find repeated Kalman filtering works well.

## Methods

**FIGURE 1:**
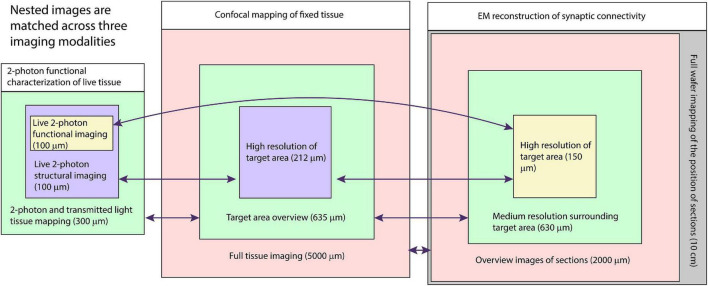
Schematic of multiresolution correlated light to EM. Nested image sets are shown for live 2-photon imaging **(left)**, confocal mapping of fixed tissue **(middle)**, and EM of synaptic connectivity **(right)**. Matched image scales are indicated by color. The high-resolution image stacks providing the functional and connectivity data for the targeted cells are highlighted in yellow. Arrows indicate which images are typically compared for matching between modalities.

Our multiresolution CLEM approach depends on acquiring a large series of images at multiple scales across multiple modalities (Figure 1). The CLEM experiment we describe in the greatest detail begins with live two-photon calcium imaging of mouse tissue. A similar approach may be used with any optical characterization that does not damage tissue ultrastructure. The key links to efficiently combining light and electron microscopy in this approach are: (1) the acquisitions of fixed tissue 3D confocal microscopy feature maps at low resolution (cell body resolution) and high resolution (subcellular features) and (2) the ability to acquire fast, low resolution, large FOV electron micrographs (cell body resolution) of sectioned tissue prior to acquiring high-resolution (cell membrane resolution) electron micrographs.

We do not quantify the precise resolution limits for each mode of imaging discussed here because the functional resolution will vary according to experiment specific details. Roughly, the “high-resolution” optical imaging in this pipeline peaks at about 0.2 × 0.6 μm for a point spread function from confocal imaging with 60 × 1.4na objective. This resolution is helpful for matching subcellular optical features such as neurites and synapses to EM reconstructions. We use a scanning electron microscope that is capable of sub-nanometer spot sizes. However, our imaging conditions balance resolving membranes with speed of imaging (more electrons = less resolution) so that our normal “high-resolution” EM spot size is about 8–15 nm across. For most image acquisitions, we follow the rule-of-thumb of choosing pixel sizes that double-sample the spot size.

All procedures in this study were approved by the Animal Studies Committee of Washington University School of Medicine (Protocols #20-190198 and #20-0055) and performed in compliance with the National Institutes of Health *Guide for the Care and Use of Laboratory Animals*.

### Live two-photon imaging

Two-photon imaging can record activity in neurons expressing the calcium sensor GcamP6. The same microscope can image structural landmarks using fluorescence or transmitted light. One of the principal concerns in using 2-photon imaging in CLEM is that extended exposure of cells to the 2-photon laser can cause tissue damage. The need for higher signal (more laser power) and stimulus repeats (number of laser exposures) must weighed against the effects of the laser on tissue ultrastructure.

**FIGURE 2:**
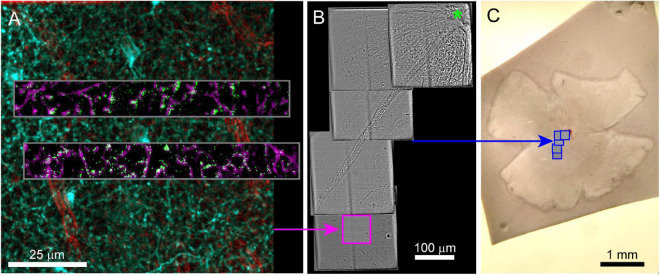
Live imaging of a retinal explant. **(A)** Two-photon imaging of calcium responses (gray inset, single frame = green, time average = magenta) to visual stimuli acquired in two rectangular ROIs and at multiple depths in the retina. Functional ROIs are aligned within two-photon structural images of the surrounding transgenic expression of the calcium indicator (2-photon) in cyan and blood vessels (scanning transmitted) in red. Blood vessels in transmitted light images are enhanced using bandpass filtering. **(B)** Scanning transmitted light images (gray, acquired on 2-photon microscope) are used to map the position of the characterized cells (pink box) relative to the optic nerve head (green asterisk). **(C)** The blue boxes map the position of the gray images on the left onto the widefield transmitted image of the entire retina.

In our example experiment, calcium responses were imaged from retinal neurons using GCamp6f (Ai148 strain, Jax #030328 crossed to Vglut3-IRES2-Cre-D, Jax # 028534). Retinas were prepared for live imaging as described in Hsiang et al. (2017). Twelve sites (two XY positions, six depths, 100 × 13 μm each) were imaged at 9.5 Hz using 930 nm two-photon excitation (Figure 2A) while visual stimuli were projected onto the retina. The overall laser intensity at the tissue was kept below 6 mW. The total functional imaging time for the 12 sites was 49 min with each site imaged for ∼3 min.

After functional imaging, 3D two-photon image stacks were acquired that encompassed the functional ROIs (Figure 2A). These larger field of view stacks (100 and 300 μm XY, 40–50 μm in depth, Table 1) provided the 3D morphology of the functionally characterized cells that is later matched to 3DEM reconstructions of the same cells. These image stacks included a channel that collected transmitted laser light. The transmitted light reveals the vasculature surrounding the functionally characterized neurons (Figure 2A). Transmitted light images were bandpass filtered using a difference of Gaussian kernel to enhance the visibility of blood vessels (see methods). After the 3D image stacks of the functionally characterized region were acquired, the target area was mapped relative to larger landmarks by acquiring a series of partially overlapping full-field (300 × 300 μm^2^) images. These images linked the target area to blood vessels, the optic disk, and the borders of the retina (Figures 2B,C). If no gross anatomical features exist that can be used to keep track of the orientation of the tissue, large-scale landmarks can be cut into the tissue either immediately before or after fixation.

### Dense feature labeling in fixed tissue

Mapping fixed tissue for CLEM using confocal microscopy has the advantage of (1) Multiple channels of information can be collected, (2) Optical sectioning enables easy 3D reconstruction, (3) Optical sectioning reduces out-of-focus light from glutaraldehyde. In widefield examinations of glutaraldehyde fixed tissue, background fluorescence can overwhelm signals from fluorescent labels and can discourage researchers from attempting optical imaging of tissue that has been fixed for electron microscopy. Confocal and two-photon imaging remove most of the out-of-focus, non-specific glutaraldehyde fluorescence leaving only the in-focus signal and background. Glutaraldehyde background signal can also be mitigated by initially fixing the tissue in lower concentrations. During initial aldehyde fixation, we used 1% glutaraldehyde and 2.5% paraformaldehyde (PFA, instead of the standard 2%/2%). For tissue that is tolerant phosphate-buffered saline (PBS), we perform our initial fixation in 0.1M PBS instead of the standard 0.1 M cacodylate buffer to reduce the exposure of microscopists to arsenic during optical mapping. After optical mapping, the tissue can be fixed again in 2% PFA, 2% glutaraldehyde in 0.1 M cacodylate buffer.

For confocal imaging, we mount tissue on a slide with a bridged coverslip for high NA oil objective imaging. Mapping can also be performed with high NA dipping cone objectives to minimize mechanical and osmotic stress on the tissue. The quality of optical maps can be improved by partially clearing the tissue in 20–47% thiodiethanol (TDE) for ∼20 min (Aoyagi et al., 2015). This treatment improves resolution and imaging depth while minimally distorting tissue (less distortion at 20%) and works well in CLEM studies (Costantini et al., 2015). Because light scatter in incompletely cleared tissue and the working distance of high-resolution oil objectives, we only acquire high resolution features from the surface (<100 μm) of the retina or brain slice. It is therefore important to map the surface that includes the cells of interest. More complete tissue clearing and/or 2-photon imaging can be used to extend the depth of high-resolution mapping.

**FIGURE 3:**
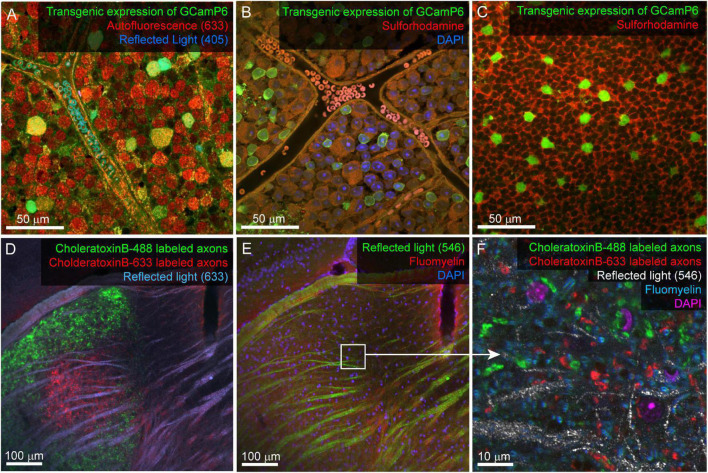
Example confocal imaging of fixed retina (top) and visual thalamus (bottom) to map features for mrCLEM. **(A)** Imaging of the ganglion-cell-side surface of a mouse retina (transgenic labeling green) with reflected 405 laser line (blue) and autofluorescence from the 633 laser line (red). Note clear cell bodies and vasculature. **(B)** Imaging of the ganglion cell layer in mouse retina (transgenic labeling green) with Sulforhodamine (red), and DAPI (blue). **(C)** Same tissue as **(B)** imaged 50 μm deeper in the inner nuclear layer. **(D,E)** Six-channel tissue mapping of a vibratome section of mouse dorsal lateral geniculate nucleus. **(D)** Retinal ganglion cell axons from the contralateral eye labeled with CtB-488 (green), and the ipsilateral eye labeled with CtB-633 (red). Fiber tracks (blue) are imaged using reflected 633 laser line. **(E)** Same image stack as **(D)**, showing DAPI (blue), FluoroMyelin (red), and 546 reflected light (green). White box indicates the field of view for **(F)**. **(F)** Single plane from a high-resolution image stack acquired at the white box in E. High-frequency matching features (FluoroMyelin = cyan, retinal ganglion cell synaptic boutons = red and green) are shown in the context of medium-resolution features (DAPI stained nuclei = magenta) and large-scale features (fiber tract = white).

Confocal mapping aims to produce a set of nested feature maps that link millimeter-scale features to micrometer-scale subcellular features. The mrCLEM features used here include (from large-scale to small-scale): (1) Gross morphology, (2) Fiber tracts, (3) Blood vessels, (4) Cell nuclei, (5) Cell bodies and neurites of labeled neurons, (6) Organelle stains (Table 2 and Figure 3).

**TABLE 2:** List of optical imaging signal sources and typical features labeled for our light to EM feature mapping.

Signal source	Biological feature
Fluorescent protein expression	Cytosolic fill of targeted neurons
Transmitted light	Gross morphology, blood vessels
Aldehyde autofluorescence	Gross morphology, blood vessels, cell nuclei
Reflected light	Gross morphology, Myelination
DAPI	Cell nuclei, chromatin distribution
Sulforhodamine	Extracellular stain (live), Heterogeneous intracellular stain (fixed), Blood vessels
Fluoromyelin	Myelin
Fluorescent Choleratoxin B	Anterograde axon tracer

A considerable amount of unlabeled 3D structural data can be obtained from most fixed tissue using intrinsic signals. The autofluorescence of aldehydes reveals neuropil and cell nuclei (by the absence of signal). We generally image autofluorescence using 633 nm wavelength laser excitation and collecting all light longer than 633 nm (Figure 3A). Reflected laser light itself can also reveal various features, including blood vessels and fiber tracts. We usually acquire a reflected light signal by scanning with the 405 nm laser line and no barrier filter (Figures 3A,D,E). The reflected light signal is biased toward surfaces parallel to the imaging plane and near the surface of the tissue. Most laser lines can be used to acquire both autofluorescence and reflected light, however, including high resolution reflected light (405 nm) and tissue penetrating autofluorescence (633 nm) adds multiscale dense features to tissue labeled with more standard red and green fluorescent markers. Six channel imaging (Figure 3D,E) is accomplished by two serial scans using three lasers simultaneously in each scan. In this example DAPI and Fluoromyelin (blue and red) are acquired simultaneously as are Alexa 488 and Alexa 633 (green and far red). Channels collecting reflected light or autofluorescence can then be added to each scan without sacrificing scan speed, signal, or emission separation.

Reflected light and autofluorescence have the advantages of being quick, easy, and universal. Increased signal and additional feature selectivity can be achieved with stains. We find a combination of DAPI and Sulforhodamine particularly effective for performing mrCLEM (Table 3). DAPI staining has the advantage of revealing the pattern of chromatin density which varies between nuclei and is visible in low resolution (<1 μm pixel size) EM. DAPI does not seem to significantly impact ultrastructure (Tarnowski et al., 1991). In live tissue, Sulforhodamine is a primarily extracellular stain that labels blood vessels, astrocytes, or damaged cells (Schlichtenbrede et al., 2009). In fixed tissue, Sulforhodamine labeling in the cytosol is more common, such as in the case of retinal ganglion cells whose axons have been cut (Figure 3B). The resulting signal reveals blood vessels, nuclei (by their lack of labeling), and changes in tissue texture such as between neuropil and nuclear layers (Figures 3B,C). Additional organelle stains can generate higher frequency features for fine CLEM alignment. Here we show six-channel imaging of a brain slice where FluoroMyelin (ThermoFisher Scientific) generates dense optical labeling of myelinated axons in the visual thalamus (Figures 3D–F).

**TABLE 3:** Protocol for combined DAPI and Sulforhodamine stain.

	0.1 M Cacodylate buffer or 0.1 M Phosphate buffer
5 min (3 times)	wash in buffer
1–24 h	300 nM 4’,6-diamidino-2-phenylindole (DAPI) in buffer. In 1 h, the stain penetrates ∼50 μm.
5 min (three times)	Wash in buffer
5 min	7 μM Sulforhodamine 101 in buffer
	Rinse once without washing out stain

### Example of multiresolution optical mapping of functionally characterized mouse retinas

**FIGURE 4:**
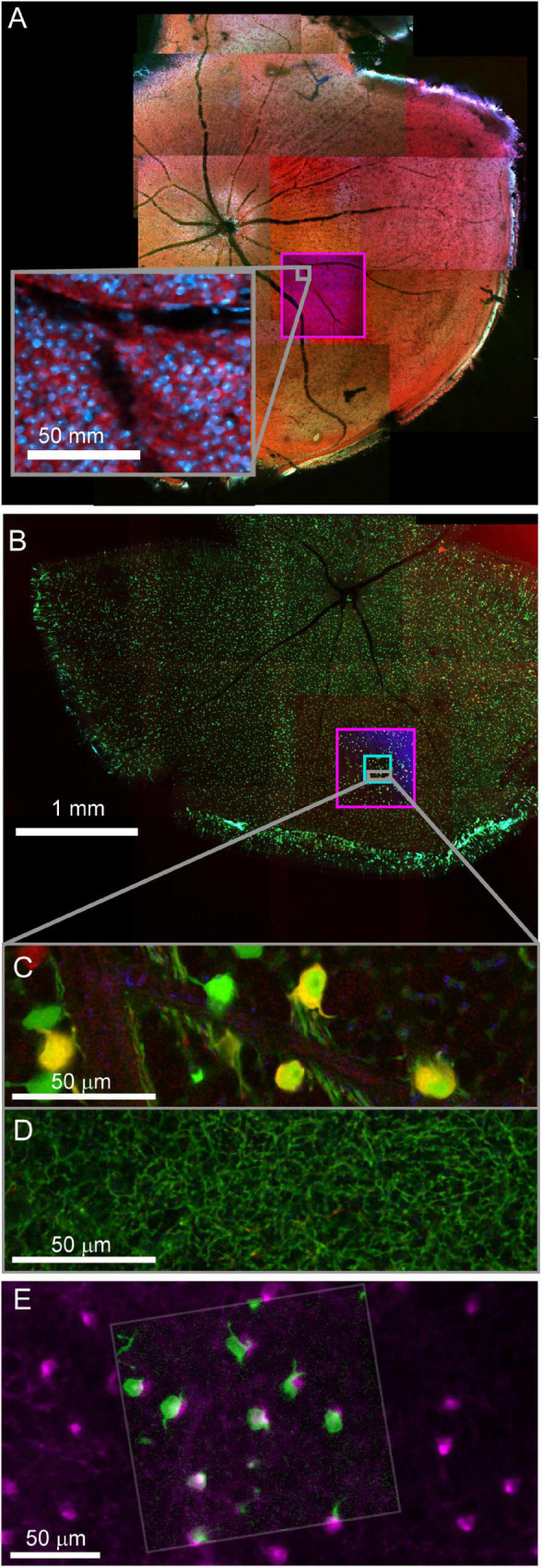
Confocal maps of fixed tissue. Low-resolution mosaics are acquired from the whole tissue, and higher-resolution image stacks are acquired that encompass targeted regions of interest. **(A)** Mosaic of mouse retina stained with Sulforhodamine (red) and DAPI (blue). Pink box indicates targeting of image stack acquired with 20x objective. The gray insert provides a closer look at the images in the pink box. **(B)** Mosaic of mouse retina transgenically expressing GCamp6 (green) and td-tomato (red). Pink and cyan boxes indicate targeting of 20x and 60x objective image stacks. **(C,D)** Two planes of one high-resolution (60x) image stack with labeled cell bodies **(C)** and neurite plexus **(D)**. **(E)** Overlay of showing the alignment of cell body signal in a 2-photon live image of GCamp6 (green) and fixed confocal image stack of GCamp6 in the same region of tissue (magenta).

Fixed optical mapping begins with imaging the entire tissue (∼4 mm wide for mouse retina) using a confocal mosaic of large field-of-view (FOV) tiles (10x objective, FOV = 1270 μm, pixel size = 0.6 μm) or wide-field epifluorescence scope. For a 150 μm thick retina, the gross morphology, blood vessels, and transgenic expression pattern could be mapped with one or a few z-planes per mosaic tile (fewer planes for gross morphology, more planes for cell bodies, Figures 4A,B). In confocal maps where transgenically labeled cell bodies are clearly visible, comparison of the fixed map to the live blood vessel map and live transgenic expression map are sufficient to identify the cell bodies of the functionally characterized neurons in the confocal map. With the position of the functional ROIs identified, image stacks are acquired encompassing the target cells; a 635 μm FOV image stack acquired at 0.3 μm resolution (20x na 0.85 objective) and a 212 μm FOV image stack acquired at 0.1 μm resolution (60x na 1.35) (Figures 4C,D). Alignment of confocal and 2-photon stacks (Figure 4E) links the fixed confocal map to functional imaging. We chose imaging parameters to provide reliable reconstructions of neurites that could later be matched to both the structural two-photon images and EM reconstructions.

**FIGURE 5:**
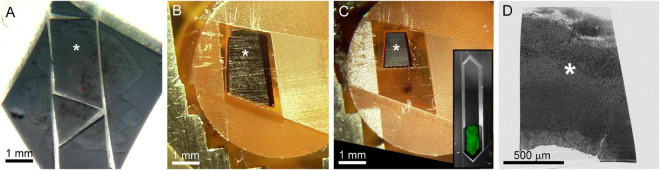
The optically characterized region of interest is excised from the aldehyde fixed tissue and processed for EM. The white asterisk tracks the region of interest across panels. **(A)** A 2 mm × 3 mm asymmetric slab is excised from a retinal whole mount. Asterisk indicates the targeted region of interest in all panels. **(B)** The tissue is stained and embedded in resin (see section “Materials and methods”). **(C)** The block is trimmed to a trapezoid approximately 800 μm × 1200 μm centered around the optically characterized region of interest. The gray inset shows how to trim a block (extended hexagon, different tissue block) for direct-to-tape automated cutting. The green channel shows that tissue features can be obtained from wide field reflected light imaging of the surface of a trimmed block face. **(D)** Overview EM image of 40 nm-thick section taken from blockface in **(C)**.

After confocal imaging, a subsection of the tissue (∼1–5 mm × 1–5 mm) encompassing the region of interest is excised for EM processing. A scalpel is used to cut an asymmetric perimeter that can readily be used to identify the orientation of the tissue. Tracking this orientation makes it possible to position the tissue in an embedding capsule so that the tissue surface closest to the cells of interest can be targeted for selective cutting (if the whole depth of the tissue block is not required for reconstruction). Widefield imaging records the position of the tissue excision (Figure 5A). Images are also acquired during the trimming of the resin block to track the position of the optically characterized neurons (Figures 5B–D). The region of interest tracked first by comparing the borders of the excised tissue before and after embedding (Figure 5A vs. 5B), then by aligning the borders of the resin block and trimming marks between stages of trimming (Figures 5B vs. 5C), and finally by recording the position of the target area relative to the final blockface borders (Figures 5C vs. 5D).

### Tissue staining for electron microscopy

A significant advantage of mrCLEM is that it does not require additional EM labeling (e.g., metal particle antibodies, tissue marking, or DAB reaction). The EM staining protocol can therefore be optimized for maximum membrane staining (osmium tetroxide + osmium tetroxide + uranyl acetate + lead aspartate, Table 4) and integrity; properties critical for high throughput 3DEM circuit reconstructions. While the same mrCLEM approach can be implemented with different types of section preparation, producing high-contrast sections on a stable substrate is ideal for our multiresolution approach.

**TABLE 4:** Generic protocol for heavy staining and resin embedding of tissue for electron microscopy.

	* All H_2_O is filtered	
2 h	1% Glutaraldehyde, 2.5% Paraformaldehyde, 2 mM Calcium Chloride	RT
3 × 5 min	wash in 0.1 M Cacodylate buffer	RT
1 h	2% Osmium Tetroxide in 0.1 M Cacodylate buffer	RT
1 h	2.5% Ferrocyanide in 0.1M Cacodylate buffer	RT
5 × 10 min	wash 0.1M Cacodylate buffer	RT
20–45 min	0.1 – 1% Thiocarbohydrazide	40 C
5 × 10 min	wash H_2_O	40 C
1 h	2% Osmium Tetroxide in H_2_O	RT
5 × 10 min	wash with 0.05 Maleate Buffer	RT
12 h	1% Uranyl Acetate in 0.05 M Maleate Buffer	4 C
4 h	1% Uranyl Acetate in 0.05 M Maleate Buffer	50 C
5 × 10 min	wash with 0.05 Maleate Buffer	RT
5 × 10 min	wash H_2_O	RT
10 min each	H_2_O to Acetonitrile dehydration 30, 70, 80, 90, 95, 100%, (freshly opened) 100%, 100%	RT
>2 h each	Acetonitrile: Resin, 3:1, 1:1, 1:3	RT
3 × 12 h	Pure Resin, rotating	RT
48 h	Polymerization in oven	60 C

The protocol presented here is used for mouse retina, but also works well for vibratome brain slices by adjusting the osmium penetration times by approximately 1 h/100 μm of penetration depth (Morgan et al., 2016).

The tissue is initially stained for 1 h in 2% osmium tetroxide (Table 4 for complete protocol). Following Hua et al. (2015), the osmium is then reduced with 2.5% ferrocyanide (ferricyanide is used in the EM images presented here but generally gives inferior results). After washout, the osmium linker thiocarbohydrazide (Willingham and Rutherford, 1984; Tapia et al., 2012) or pyrogallol (Mikula and Denk, 2015; Genoud et al., 2018) is used to bind a second layer of osmium to the first. The tissue is then treated with the second osmium stain, uranyl acid, and lead aspartate. Post-section staining with lead citrate (Morgan et al., 2016) can replace the lead aspartate staining to improve ultrathin sectioning and synapse labeling. The tissue is dehydrated in acetonitrile and embedded in Epon-812, Spurrs, or LX-112. In our experience, Epon-812 is reliable, but can produce large compression differences between tissue and non-tissue regions of a blockface (leading to wrinkles). Spurrs and LX-112 (Wanner and Vishwanathan, 2018) tend to produce fewer wrinkles.

### Tissue sectioning with automated tape ultramicrotomy (ATUM)

Ultrathin sections are collected on conductive tape using the ATUM (Schalek et al., 2012; Hayworth et al., 2014; Baena et al., 2019). Tissue blocks are first trimmed either into a standard trapezoid shape or an extended hexagon (Morgan et al., 2016). Ideally, a trapezoid should be about 1.5 mm tall so that each section deposits the preceding section on the collection tape. Especially in the case of blockfaces wider than 1 mm, an extended hexagon face with a leading and trailing point can help with cutting reliability. The hexagonal block face must be long enough (>3 mm) so that the section is picked up by the collection tape as it finishes cutting the section. For the retinal tissue, 40 nm thick sections were collected at 0.2 – 0.4 mm/second cutting speed. Thinner sectioning of a large section series is possible (10,000 sections at 30 nm, Morgan et al., 2016), but obtaining reliable sectioning is significantly more difficult below 40 nm and section quality degrades below 20 nm. Thicker sectioning can be appropriate for some tissue, but as sections become thicker, the range of membrane angles and neurite diameters that introduce reconstruction ambiguities increases. As most of the fine processes we are reconstructing in the mouse retina are ∼100 nm diameter or larger, 40 nm section thickness provides a good balance of sampling frequency and sectioning reliability. In our experience, a fresh patch of a diamond knife can cut through approximately 3 m of heavily stained tissue (3000 sections of a 1 mm tall piece of tissue from a > 1mm wide block face) before knife wear prevents lossless ultrathin sectioning. Performance varies with the density and distribution of metal in the block.

During ultrathin sectioning, the water level in the knife boat is maintained automatically. Monitoring can be performed using software provided by Powertome or with our customizable Matlab water monitoring software^1^.

After collecting sections, the tape is cut and permanently attached to 10 cm wide silicon wafers using double sided vacuum-safe carbon tape (Schalek et al., 2012). The conductive surface of the Kapton tape is grounded by bridging all of the tape surfaces to each other and to the silicon wafer with millimeter wide strips of conductive carbon tape. Wafer mounted sections (∼100–200 per wafer) can be stored for years and reimaged many times (Hildebrand et al., 2017; Liu et al., 2021). The primary limit to reimaging is that the sections can be damaged if intensive imaging interacts with contaminated surfaces. Allowing sections to outgas under vacuum for several hours or plasma cleaning the surface of the sections reduces this damage.

### Electron micrograph tissue mapping

Wafers containing ultrathin sections are mapped using the custom Matlab package WaferMapper as previously described (Hayworth et al., 2014^2^, Table 1). Section mapping and automated imaging can also be achieved using the array tomography software available with the Zeiss Merlin/Atlas system. Here, we use WaferMapper due to its customizability. For both accurate targeting and stitching tiles together, it is important that pixel positions accurately reflect stage positions. We, therefore, run a pixel-to-stage calibration between any change of imaging conditions (voltage, stage position, FOV) and data acquisition.

**FIGURE 6:**
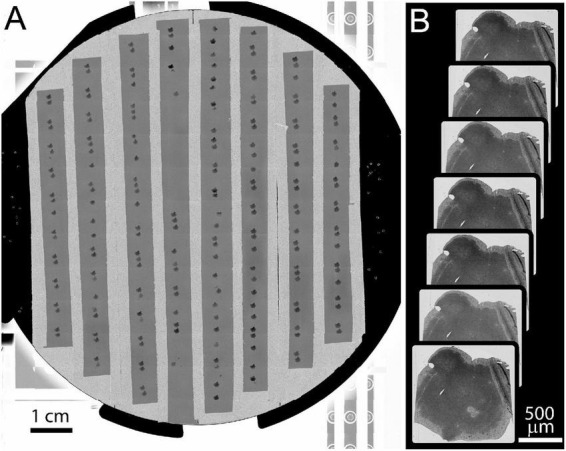
Example of full wafer image and section overview images. **(A)** Full wafer EM image of the 10 cm wafer is acquired as a 14 × 12 tile mosaic. **(B)** An overview image is acquired for each section. These overviews are then aligned to a template image to generate a 3D volume of the entire tissue.

Image mosaics of the whole 10 cm wafer were acquired in the Merlin SEM using mosaics of 8 × 8 mm tiles at 8 μm pixel size (Backscatter detector, BSD). The full-wafer images are used to identify the positions of ultrathin tissue sections (Figure 6A). These section positions are then used to direct the automatic acquisition of section overview images (FOV = 3072 μm, pixel size = 0.75 μm, Figure 6B). The overview images can be acquired with the BSD detector (minimum imaging artifacts) or SE2 detector (fast). The overview images are then aligned to form a 3D map of the collected sections. Additional image stacks are acquired by defining points of interest within the aligned overview image volume.

**FIGURE 7:**
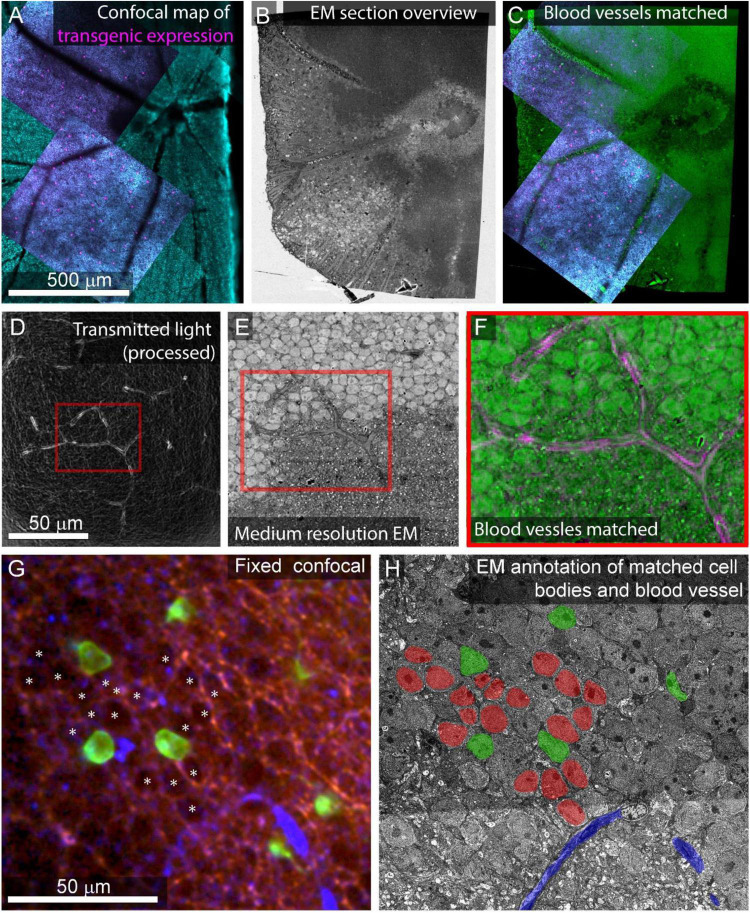
Feature matching between optical images and low-resolution EM. **(A)** Multiscale confocal maps of transgenic expression (magenta) and DAPI (cyan) in fixed tissue. Note blood vessels visible by absence of staining. **(B)** Same tissue as **(A)**. Section overview image (1 μm pixel size) of ultrathin section used to map tissue position on collection wafer. **(C)** Alignment of blood vessels between the confocal map from A (blue, red) with the section overview from **(B)** (green). **(D)** Transmitted light image of live retina filtered with edge detection to show blood vessels. Red box corresponds to the position of red box in **(E,F)**. **(E)** Electron micrograph of tissue shown in **(D)** acquired at 40 nm pixel size. Note the blood vessel in the red box. **(F)** Alignment of the blood vessel shown in red boxes in panel **(D)** (magenta) and E (green). **(G)** Confocal stack of fixed tissue showing transgenically targeted cells expressing fluorescent protein (green), positions of surrounding cell nuclei (white asterisk) visible by the absence of autofluorescence (red), and blood vessels visible by reflected light (blue). **(H)** Medium resolution (40 nm pixel size) EM section in which the blood vessel (blue), transgenically targeted cells (green), and surrounding nuclei (red) have been matched to the confocal stack in **(G)**.

Gross morphology and blood vessels can readily be identified in the 3D alignment of overview images (Figures 7A–C). The EM images of these features can be matched to optical images of the same features. Manual matching of a small set of features by viewing the light and EM image stacks side by side (a few hours in ImageJ) is sufficient for picking ROIs for the next stages of higher resolution image acquisition.

Depending on the tissue and the quality of the overview imaging, it may be possible to identify cell nuclei well enough in the overview images to generate a nuclei-to-nuclei matching between the EM and optical maps. Otherwise, an additional medium-resolution EM image stack (FOV 630 μm, pixel size = 20 nm) can be targeted to the region of interest to align light and EM at the cell body level (Figures 7D–F). We match individual cells in light and EM images iteratively. In retinal tissue, we begin with matched blood vessels, then match nearby cell nuclei, then the nuclei close to those, continuing until we reach the cells of interest (<8 h, Figures 7G,H). Despite the structural uniformity within retinal layers, this method makes it relatively straightforward to identify the nuclei of optically characterized neurons in EM section space prior to high-resolution imaging. In other tissues, non-uniform features like myelinated axons tracts can make low resolution matching faster.

Once the relevant blood vessels, fiber tracts, and cell bodies have been matched between optical and EM tissue volumes, high-resolution image stacks (4 nm × 4 nm) can be targeted to individual optically characterized cells. For our high-resolution imaging conditions, the most efficient image tile size on our system is usually around 80 μm wide. Larger tiles reduce overlap in 2D mosaics and save tile to tile time, but image quality degrades with distance from the center of the FOV. Mosaics of these image tiles can be arbitrarily large, although automatic refocusing can become necessary for image tiles more than a hundred micrometers away from one another.

The critical limit to acquisition size for the single beam system is pixel rate. The Merlin SEM can acquire up to 20 million pixels per second (Morgan et al., 2016), however achieving enough signal to support these short dwell times is difficult, and image quality is likely to fall off at speeds greater than 5 million pixels per second. The limited pixel rate is made up for, to some extent, by the ease with which the single-beam SEM can reimage an area at a range of resolutions. Aside from being used to match optical features to EM, medium-resolution stacks (8–40 nm) can be used to trace relatively large features (such as large dendrites and myelinated axons) out of high-resolution volumes and across volumes to large to be imaged at high-resolution.

### Image processing

For some optical images, we use median filters to reduce noise, FFT bandpass filters (ImageJ) or difference-of-Gaussian filters (Matlab) to enhance signals at select frequencies, and/or edge detection (ImageJ) to enhance blood vessels. Frequency enhancement is particularly useful in emphasizing blood vessels in transmitted light images and enhancing cell nuclei in images with very low signal. For difference-of-Gaussian filtering, the size of the central Gaussian is chosen to match the size of the feature of interest (sigma ∼5–10 μm, for blood vessels and cell bodies). The subtracted surround was a Gaussian with a larger sigma. We tuned kernel sizes empirically.

To reduce noise in many of our electron micrographs, we median filter the images using a 3 × 2 kernel. We use a kernel that is smaller in the X dimension, because there can already be some signal spread along the X dimension when scan speed nears the frequency response limit of the detection system (∼10 MHz). We then normalized the brightness and contrast of each image section to match the mean and range of intensities between images. Image stacks are registered using TrakEM2 within FIJI (ImageJ) using a SIFT-based rigid registration, followed by affine registration, manual landmark-based correction, and finally elastic registration (ScalableMinds, Saalfeld et al., 2012). Cells are then annotated by manual tracing using VAST^3^ (Berger et al., 2018).

### Alignment of neurites between light and electron microscopy

**FIGURE 8:**
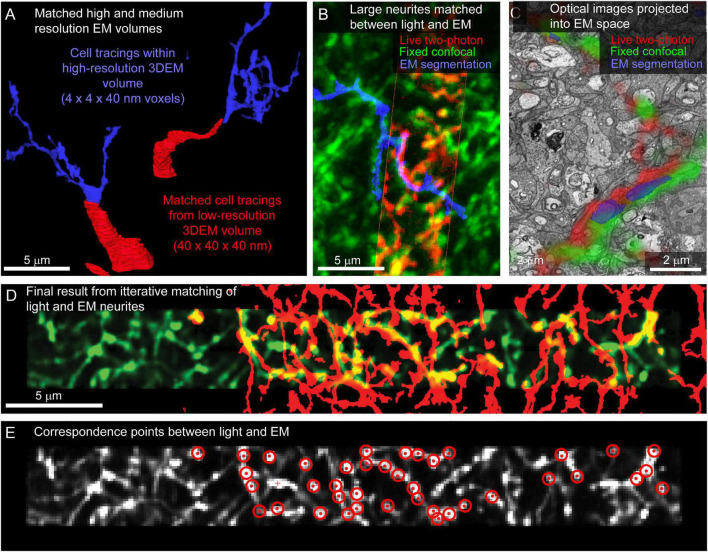
Matching neurites between optical images and EM. **(A)** The proximal neurites (red) of cell bodies identified in a large field, medium resolution image volume (40 × 40 × 40 nm voxel size, Figure 5H) are traced into the high-resolution image volume (blue, 4 × 4 × 40 nm voxel size). **(B)** Matching the morphology of EM reconstructed neurites (blue) to images from fixed (green) and live (red) optical imaging confirms the initial matching of cell bodies. **(C)** Single slice from EM volume (viewed in VAST) where live two-photon (red) and fixed confocal (green) fluorescence images of the neurons of interest are aligned with the EM volume to determine neurite-to-neurite matches with EM traced (blue) neurons. **(D)** Optical image (red) affine transformed to better fit the EM traced neurites (blue). **(E)** Correspondence points (red targets) where positions in the optical image (gray) have been mapped onto positions of EM traced neurites.

For tissue where fine-scale features (such as neurites) are connected to cell bodies within the high-resolution EM volume, the initial link between cell body identification and matching of smaller features is straightforward. In the example retina dataset, neurites are reconstructed by tracing the primary neurites from the cell body located in medium resolution EM volumes into high-resolution EM volumes (Figure 8A). Cell matching and tracing accuracy is confirmed by superimposing 3D renderings of optical images and EM segmentations using Amira (ThermoFisher Scientific) (Figure 8B).

Once the target cells are reconstructed, the ease of matching subcellular features within an arbor depends on the structural details of the neurons and the quality and sparseness of the optical maps. For the live retinal imaging example, optical labeling of the neuropil was too dense for most neurites to be matched using a manual side-by-side comparison of the light and EM images. To align optical data and EM segmentations at the micrometer scale, we use matched fiducial points from cell nuclei and large neurites to calculate an affine transformation of the optical data into the EM volume space (ImageJ). The matching is further refined using a thin plate spline transform with additional fiducial points. We then are able to view the optical images superimposed on the raw EM data and EM segmentations in VAST (Figure 8C) where we can identify additional fine correspondence between optical images and traced neurites. By iteratively adding more tracing, more correspondence points, and re-transforming the optical data, we generate a dense mapping of correspondence between the optical and EM images (Figures 8D,E).

The fine-scale projection of the optical data into the EM volume also allows us to identify optically characterized neurites that were not previously traced in the EM volume. Within a plexus of labeled neurites (Figures 1A,B), we could iteratively (1) identify a correlated neurite, (2) determine where the next closest optically imaged neurite should be, and (3) perform dense neurite segmentation in the projected region to find the neurite with morphology matching the optical image. Leapfrogging through the optically imaged plexus is significantly more difficult than matching neurites from labeled cell bodies. The general approach to matching light and EM neurites is described in detail by Drawitsch et al. (2018), and the efficiency of the approach is aided by starting with a saturated segmentation of all neurites in the region of interest.

**FIGURE 9:**
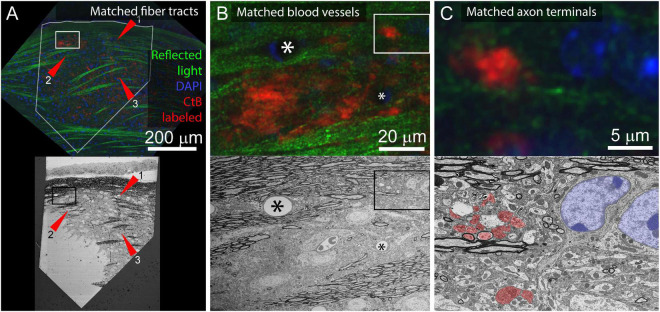
Application of multiresolution feature matching in a brain slice. **(A)** The initial alignment of light and EM uses myelinated fiber tracts. Top panel shows confocal image of aldehyde fixed dLGN coronal slice. Red arrows indicate three myelinated tracts that are visible in both reflected light and EM. Green = reflected light, Blue = Nissel stain, Red = axon terminals of CtB injected retinal ganglion cells. Bottom panel shows EM image of the surface of the same brain slice. The white outline in the optical image indicates the position of the EM section. Rectangles indicate the position of images in **(B)**. **(B)** Secondary alignment uses blood vessels. Top and bottom panels are higher resolution image acquisitions from positions shown in **(A)**. Corresponding blood vessels are indicated by asterisks. Rectangles indicate the position of **(C)**. **(C)** Cropped images from **(B)** showing correspondences of synaptic bouton and chromatin signals. In the EM image, retinal ganglion cell boutons (ultrastructurally identified by light mitochondria) are highlighted in red and match with the CtB labeled boutons in the optical image. The nucleus and chromatin pattern in EM are highlighted in blue and corresponds to the DAPI labeling in the optical image.

For tissue where the cell bodies of the neurites of interest are not included in the sectioned volume, it is possible to match features between scales using other dense correlation features. For example, to identify contralaterally projecting retinal ganglion cell boutons in the lateral geniculate nucleus, reflected light imaging of fiber tracts (low and medium-resolution features), DAPI staining (medium and high-resolution markers), and fluorescently tagged Cholera Toxin B (CtB) labeling of the axons of interest was sufficient (Figure 9). This application benefits from the distinctive ultrastructural profile of retinal ganglion cell boutons.

## Results

**FIGURE 10:**
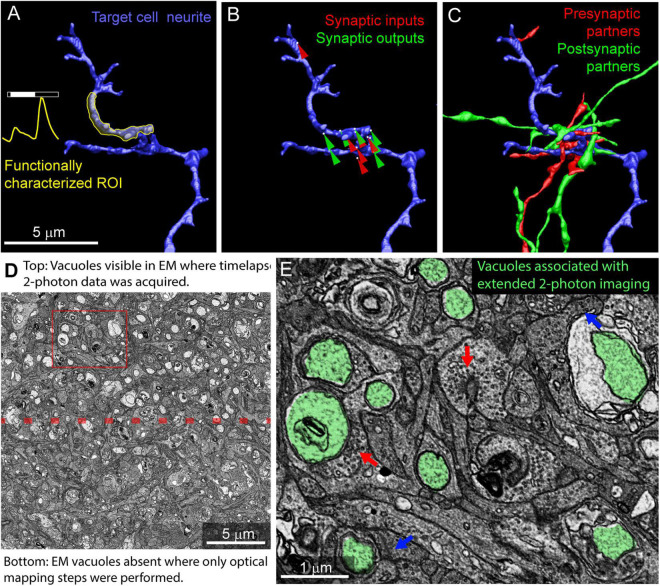
Example results from multiresolution matching of functional imaging and EM. **(A)** Local neurite responses (yellow) are mapped onto EM reconstructions of targeted neurons (blue). Same neurite as in Figure 8B. **(B)** Synaptic inputs (red) and outputs (green) are mapped on the neurite of interest. **(C)** Pre (red) and postsynaptic (green) cells synaptically connected to the neurite of interest are reconstructed. **(D)** Vacuoles appear in regions of EM tissue that have been imaged continuously with two-photon calcium imaging (top) but are uncommon in the surrounding, optically mapped tissue (bottom). The red box indicates the region shown in **(E)**. **(E)** Ribbon synapses (blue arrows) and conventional synapses (red arrows) are still identifiable in the two-photon damaged tissue. Vacuoles are highlighted in green.

Using the mrCLEM approach described above, it is possible to selectively image and reconstruct the synaptic connectivity of optically characterized neurons and neurites (Figures 10A–C). The time devoted to correlating light and EM using this approach is small relative to the time required to reconstruct circuits with EM. Acquiring the confocal images required to map a fixed piece of tissue can be completed in less than 12 h (Table 1). Acquisition of the low- and medium-resolution EM images to be matched to the confocal maps requires 1 week (hundreds of sections) to several weeks (tens of thousands of sections). Manually matching of features linking the confocal and EM images may take several days. By contrast, the high-resolution imaging of multi-terabyte EM datasets can take months and generates a dataset that can be mined for years. Figure 10C shows a cutout of a larger reconstruction of the functionally characterized cells in which synaptic inputs and outputs are identified and the synaptic partners of the functionally characterized neurites have been reconstructed.

The accuracy of selective imaging is limited by several processes. First, when wafers are reloaded into the microscope for new imaging sessions, slight differences in wafer position must be compensated for by comparing new images of fiducial points on the wafer to previous images. While this process can produce accurate (within ∼10 μm) targeting of previously defined feature positions, there are also many sources of potential error that can result in mistargeting (∼100 μm shift). To circumvent these reloading errors, a final round of image-based stage correction compares the current view of the targeted region of interest to the previously imaged and aligned overview images. Given a good alignment of these overview images and clear low-resolution features (such as cell bodies) this second targeting process can produce accurate cell-level automated image targeting. The current implementation produces a median section to section displacement of 1.08 μm (100 sections measured, max = 6.22 μm, 95% CI = [0.17 – 2.74 μm]).

Photodamage is a potential consequence of mapping tissue using repeated optical imaging. We have not observed ultrastructural damage associated with Sulforhodamine labeling, DAPI labeling, confocal mapping of fixed tissue, or two-photon structural mapping of live tissue. We have, however, observed ultrastructural signs of damage in regions of tissue where live calcium imaging was used to characterize neurite responses. In regions of the tissue subjected to two-photon imaging for extended periods of time, large pale vacuoles are common in the cytosol (Figures 10D,E). Most of these vacuoles are well contained within the cytosol of the cells and do not disrupt tracing. Some vacuoles are large enough to exclude surrounding cytosol, thereby increasing tracing ambiguity. Synapses within the heavily imaged region appeared ultrastructurally normal (Figure 10E). We conclude that, while photodamage must be monitored in mrCLEM experiments, optical mapping steps can be performed at exposure levels conducive to circuit reconstruction.

## Discussion

The multiresolution CLEM (mrCLEM) approach we describe here provides a practical and relatively low-cost path forward for combining connectomic, functional, and molecular data. Multiresolution approaches to CLEM can be executed using a variety of technologies. Here, we highlighted the advantages of our ATUM/SEM approach: (1) Sections mounted on ATUM collection tape can be imaged repeatedly without distortion. (2) Modern SEMs can readily switch between millimeter-scale fields of view and nanometer-resolution imaging and therefore are well adapted to automatically mapping and imaging large numbers of sections. (3) The easy generation of micrometer resolution 3DEM volumes makes mrCLEM through pattern matching relatively easy, thereby permitting optimal EM staining. (4) Optical imaging of dense features such as autofluorescence, reflected light, organelle stains, and non-specific stains provides excellent features for matching to medium-resolution EM maps.

The imaging parameters provided here are meant to communicate the range of scales and modalities that can be linked together in the examination of a single piece of tissue. The specific parameters will vary from experiment to experiment. The most important factor to consider when replicating this multiresolution CLEM pipeline is the ease with which EM images can be acquired from the same tissue at multiple resolutions. Ideally, the electron microscope used should support being able to quickly acquire overview images that are several millimeters wide in which cell bodies can be resolved. The second critical issue is identifying the high-resolution features that can be both labeled at a useful frequency (not too sparse or dense) in optical images and that can be efficiently annotated in the EM images.

In general, the mrCLEM approach presented here applies to a wide range of tissues because it depends on generic tissue features and labeling. Our example of retinal explants comes with both advantages and disadvantages. The flatness of the retina meant that all cells of interest were readily accessible with confocal microscopy. Performing CLEM on flat tissue also helped to ensure that the light and EM sectioning planes were parallel, thereby eliminating two degrees of freedom from the matching process. Both advantages can also be obtained in brain slices (<1 mm thick), although tissue clearing and/or two-photon imaging might be required for thicker slices. The most crucial advantage of performing CLEM on retinal tissue was the proximity of the neurites of interest to their cell bodies. This proximity allows for easy linking between medium- and high-resolution matching features. At the same time, the uniformity of cells and neurites within retinal layers and the lack of myelinated axons make pattern matching in the retina more difficult than pattern matching in slices of the lateral geniculate nucleus.

If neurites of interest are connected (within a hundred micrometers or so) to large neurites or cell bodies that can be traced at lower resolution, the multiresolution CLEM approach is efficient. The most significant limit of the mrCLEM approach occurs when thin neurites of interest are not connected to any nearby large neurites (visible in medium resolution EM), myelin, the cell body, or other larger structure. This case requires crawling through dense labeling of optical features from one correlated feature to the next projected correlation. If hundreds of fine neurites need to be reconstructed by this leapfrogging, the efficiency of mrCLEM targeted reconstruction relative to dense reconstruction of all structures is reduced. In cases where easy linking between medium and high-resolution volumes is not available, and dense reconstruction of large volumes is not required, CLEM through label matching such as NIRB or peroxidase labeling is likely to be a more efficient solution.

Advances in the speed with which serial section EM volumes can be acquired and analyzed have the potential to make major contributions to neuroscience. One application of these advances is the acquisition of petabyte-scale datasets that provide complete descriptions of the organization of important circuits. A parallel path for developing high-throughput volume EM is for terabyte-scale EM volumes to become integrated with the rest of neuroscientific data collection. For this path to be successful, 3DEM connectomic data will regularly be paired with other data modalities, and multiple EM volumes will be acquired for each experimental condition. Multiresolution CLEM, by merging data modalities and targeting connectomic reconstructions to cells and neurites of interest, can accelerate this process.

## Data availability statement

The raw data supporting the conclusions of this article will be made available by the authors, without undue reservation.

## Ethics statement

The animal study was reviewed and approved by Animal Studies Committee of Washington University School of Medicine.

## Author contributions

JM, DK, KF, and PR contributed to the conception and design of the study. J-CH performed the live imaging. KF performed the optical mapping and EM imaging of functionally imaged tissue. PR obtained the optical maps with fluorescent dyes. KV prepared the tissue for EM. All authors contributed to manuscript revision, read, and approved the submitted version.

## Footnotes

1
https://github.com/MorganLabShare/mrCLEM2021

2
https://github.com/MorganLabShare/WaferMapper

3
https://lichtman.rc.fas.harvard.edu/vast/
